# Clinicopathologic Findings in Fatal Neurotoxicity After Adoptive Immunotherapy With CD19-Directed CAR T-Cells

**DOI:** 10.1097/HS9.0000000000000533

**Published:** 2021-02-10

**Authors:** Philipp Karschnia, Felix Strübing, Nico Teske, Viktoria Blumenberg, Veit L. Bücklein, Christian Schmidt, Florian Schöberl, Konstantinos Dimitriadis, Robert Forbrig, Hans-Joachim Stemmler, Joerg-Christian Tonn, Michael von Bergwelt-Baildon, Marion Subklewe, Louisa von Baumgarten

**Affiliations:** 1 Division of Neuro-Oncology, Department of Neurosurgery, Ludwig-Maximilians-University School of Medicine, Munich, Germany; 2 German Cancer Consortium (DKTK), Partner Site Munich, Germany; 3 Center for Neuropathology and Prion Research, Ludwig-Maximilians-University School of Medicine, Munich, Germany; 4 Department of Medicine, Hematology & Oncology Division and Cellular Immunotherapy Program, Ludwig-Maximilians-University School of Medicine, Munich, Germany; 5 Laboratory for Translational Cancer Immunology, Gene Center of the Ludwig-Maximilians-University Munich, Munich, Germany; 6 Department of Neurology, Ludwig-Maximilians-University School of Medicine, Munich, Germany; 7 Department of Neuroradiology, Ludwig-Maximilians-University School of Medicine, Munich, Germany.

Chimeric antigen receptors (CARs) incorporate antigen-recognition moieties that endow an autologous polyclonal T-cell population with major histocompatibility complex-unrestricted specificity against a tumor cell-antigen such as the B-cell-antigen CD19. Several clinical trials have shown high response rates for immunotherapy with CD19-directed CAR T-cells in patients with large B-cell lymphoma,^1^ B-cell lymphoblastic leukemia,^2^ and mantle-cell lymphoma.^3^ However, broader success of CAR T-cell therapy is hampered by unique adverse effects including cytokine release syndrome (CRS) and neurotoxicity which is also denoted by the term “immune effector cell–associated neurotoxicity syndrome” (ICANS). Whereas CRS is a well-characterized phenotype mimicking sepsis and therapeutic agents are available to alleviate symptoms, pathogenesis and management of neurotoxicity are less well understood. Although considered a rare event, fatalities in the setting of neurologic symptoms following CAR T-cell infusion have been described.^4^ Data on neuropathological findings are therefore limited. We present a case of fatal neurotoxicity after treatment with CD19-directed CAR T-cells, outline neuropathological findings, and interpret our findings in light of novel pathogenetic models of ICANS (particularly focusing on evidence for cross-reactivity of CAR T-cells against brain mural cells in our case).

A 65-year-old woman presented to our service for evaluation of CAR T-cell therapy for refractory diffuse large B-cell lymphoma (DLBCL). Four years before, initial diagnosis of DLBCL stage IV was made and 5 lines of chemotherapeutic regimens were provided within the following years but disease eventually progressed. Sites of disease manifestation at presentation to our service included mediastinal as well as axillary lymph nodes and malignant pleura effusions. Karnofsky performance score was 90. Neurologic examination, electroencephalography (EEG), cerebrospinal fluid (CSF), and brain magnetic resonance imaging (MRI) were unremarkable and ruled out cerebral disease involvement. Following lymphodepletion with cyclophosphamide (500 mg/m^2^) and fludarabine (30 mg/m^2^), CD19-directed CAR T-cells (axicabtagene ciloleucel; 2 × 10^6^ CD3^+^-cells/kg body weight) were administered intravenously (Figure 1).

**Figure 1.**
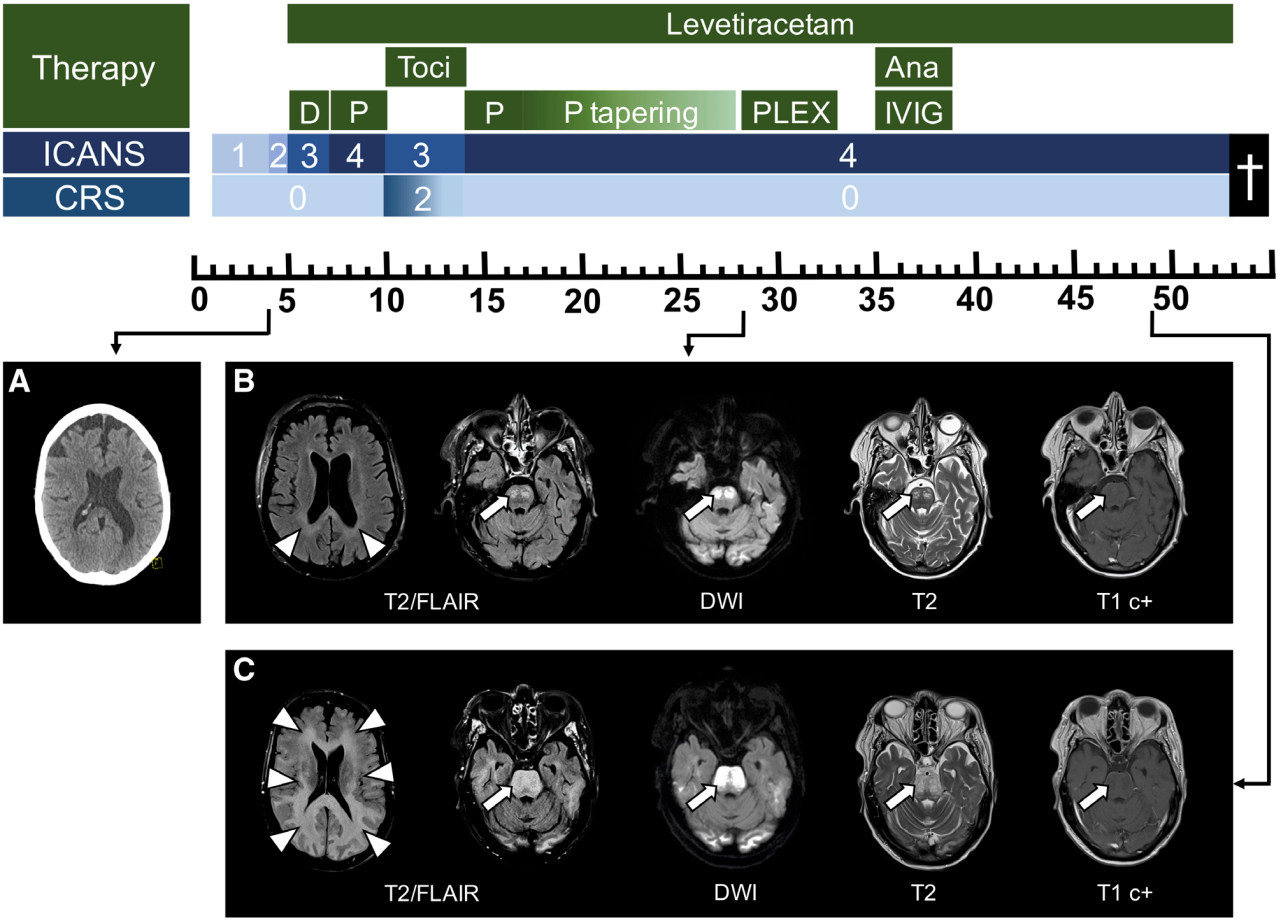
**Timeline and management of neurotoxicity following CAR T-cell therapy.** Upper part: Swimmer plot depicting the kinetics and management of neurotoxicity through 55 d after CAR T-cell infusion. The highest grade of neurotoxicity (ICANS; navy blue row), CRS (light blue row), and provided therapy (green rows) on each day are color-coded. Imaging studies are also indicated. (A), Computed tomography of the brain at onset of ICANS grade 2 showing unspecific frontal atrophy without intraaxial pathologies. (B), Axial T2/FLAIR- (left panel), DWI- (middle left panel), T2- (middle right panel), and T1-postcontrast (right panel; T1 c+) weighted MRI of the brain during ICANS grade 4 demonstrating symmetric white matter lesions extending along the posterior horn of the lateral ventricles (arrowheads) to the basilar part of the pons (arrows). Associated restricted diffusion indicating blood-brain-barrier dysfunction is present, whereas no contrast enhancement can be seen. (C), Axial T2/FLAIR- (left panel), DWI- (middle left panel), T2- (middle right panel), and T1-postcontrast (right panel; T1 c+) weighted MRI of the brain after several weeks of ICANS grade 4 displaying progression of the juxtaventricular supra (arrowheads) and pontine infratentorial white matter lesions (arrows). Severe pontine swelling with extensive diffusion restriction is shown, indicating profound edema of the brain stem due to blood-brain-barrier disruption. Ana = anakinra; CAR = chimeric antigen receptor; CRS = cytokine release syndrome; D = dexamethasone; DWI = diffusion-weighted imaging; FLAIR = fluid-attenuated inversion recovery; ICANS = immune effector cell–associated neurotoxicity syndrome; IVIG = intravenous immunoglobulins; MRI = magnetic resonance imaging; P = prednisolone; PLEX = plasma exchange; Toci = tocilizumab.

Daily screening for neurotoxicity was done using the Immune Effector Cell–associated Encephalopathy-score (ICE-score; scale: 0 points [unarousable]–10 points [normal cognition]).^5^ Following the day after CAR T-cell infusion, the patient had mild dysgraphia (ICE-score 9/10; ICANS grade 1). Four days after CAR T-cell infusion, the patient started experiencing moderate disorientation and agitation (ICE-score 6/10; ICANS grade 2). Lumbar puncture and cranial computed tomography (CT) performed at that time revealed normal findings. One day later, neurologic symptoms deteriorated and the patient was progressively disorientated and difficult to arouse (ICE-score 2; ICANS grade 3). EEG revealed generalized periodic discharges; levetiracetam (1000 mg q12h) and dexamethasone (10 mg q6h) were started. However, symptoms further progressed and the patient was transferred to the intensive care unit (ICU) 6 days after CAR T-cell therapy.

On ICU arrival, the patient was stuporous (ICE-score 0; ICANS grade 4). A mildly increased muscle tone and a left-sided Babinski sign were appreciated on neurologic examination. EEG was consistent with delta coma; repeated head CT and lumbar puncture were unrevealing. No electrolyte imbalance necessitating significant therapeutic interventions was encountered. Protective endotracheal intubation was performed; steroids were increased (prednisolone 1 g q1d for 3 days). Following steroid treatment, the patient awakened to tactile stimuli between day 10 and 14 after CAR T-cell infusion (ICE-score 0; ICANS grade 3). During these days, the patient concomitantly developed CRS (American Society for Transplantation and Cellular Therapy grade 2) as characterized by cardiac, renal, and liver affection; but CRS quickly regressed after tocilizumab was administered. However, level of consciousness eventually decreased irreversibly after day 14. This was refractive to another course of high-dose steroids (prednisolone 1 g q1d for 3 days, followed by 1 mg/kg q1d). CSF analysis on day 23 was consistent with blood-brain-barrier disruption as indicated by elevated protein levels of 90 mg/dL. Brain MRI on day 28 after CAR T-cell infusion showed bilateral symmetric lesions in the white matter bordering the posterior horn of the lateral ventricle and also anterior to the splenium of the corpus callosum on T2/fluid-attenuated inversion recovery-weighted imaging. These lesions extended into the basilar part of the pons where associated restricted diffusion was displayed. Given the continuous steroid-refractory coma, 5 cycles of plasmapheresis were initiated between day 28 and 33 after CAR T-cell infusion. No significant clinical improvement was seen, and intravenous immunoglobulins (cumulative 2 g/kg) as well as interleukin-1 blockade using anakinra (100 mg q6h) were provided on days 35-39 which also did not ameliorate symptoms. Brain MRI revealed further progression of the lesions with profound infratentorial brain edema. Goals of care were changed, and the patient deceased 53 days after CAR T-cell infusion. Consent from the patient and, after patient’s death, from her family was obtained. All data were kept anonymously and are available on qualified request.

Autopsy was performed (Figure 2). On macroscopic examination, the brain showed occipital as well as infratentorial swelling which was accompanied by multiple intraparenchymal hemorrhages particularly pronounced in the area of the pons where lesions were also seen on imaging. On microscopic examination of pontine tissue, enlarged perivascular spaces with fluid extravasation consistent with blood-brain-barrier dysfunction were seen. Substantial accumulation of intramural and perivascular CD3^+^/CD8^+^—but also CD3^+^/CD4^−^/CD8^−^—lymphocytes was found, and polymerase chain reaction (PCR) of dissociated pontine tissue detected the presence of CAR T-cells. Within the pontine white matter, axonal injury with vacuolization and spheroid formation was also displayed and accompanied by microglia activation. There was no evidence of central nervous system (CNS) infection, and findings were not consistent with central pontine myelinolysis. Examination of the other organs showed complete lymphoma regression.

**Figure 2.**
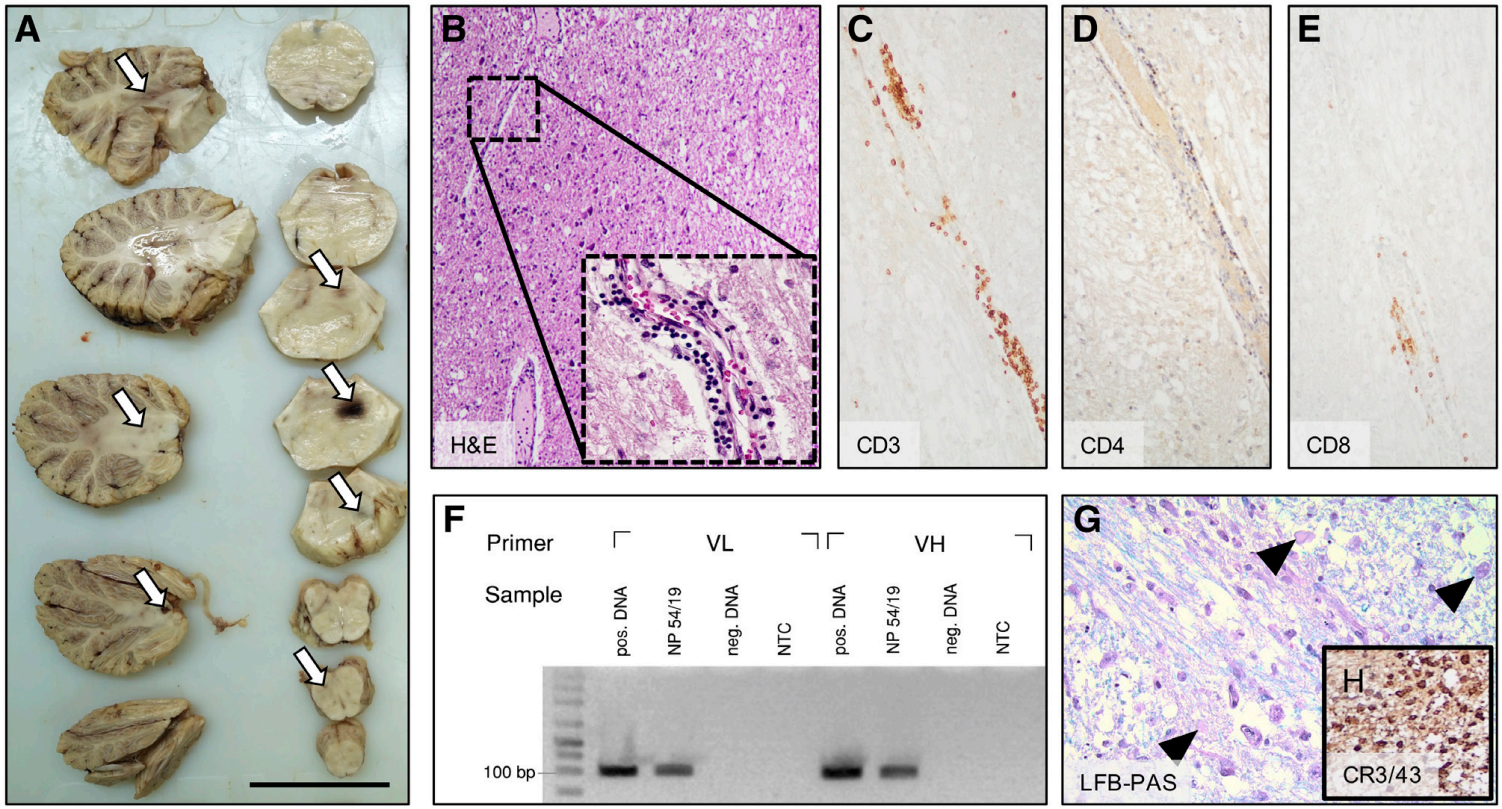
**Neuropathological findings in fatal neurotoxicity following CAR T-cell therapy.** (A), Gross examination revealing edema with markedly increased total brain weight (1459 g; normal 1250-1400 g) which is accentuated at the brainstem at the level of the pons as demonstrated by increased pontine diameter (40 mm; normal 20-25 mm). Numerous intraparenchymal hemorrhages of different size can be seen particularly within the pons, but also within the cerebellar white matter (arrows). Scale bar indicates 5 cm. B-E, H&E (B), CD3 (C), C4 (D), and CD8 (E) stains of a tissue specimen taken from the pontine area (original magnification in [B]: ×4; in [B] [inset]: ×20; in [C-E]: ×10). H&E shows dilated perivascular spaces with infiltration of inflammatory cells embedded into a loose eosinophilic matrix consistent with fluid extravasation. Immunohistochemical staining for the pan-T-cell marker CD3 characterizes the perivascular inflammatory cells as T-leukocytes which partly stain positive for CD8 but negative for CD4. Note that a number of CD3^+^ cells stain double negative for CD4/CD8. (F), Polymerase chain reaction for the light chain (VL) and heavy chain (VH) of the anti-CD19 (FMC63) CAR highlighting the presence of CAR T-cells within pontine tissue (sample NP 54/19; pos. DNA, neg. DNA, NTC). (G) and (H), LFB-PAS staining demonstrating vacuolization and axonal spheroids (arrowheads) within the pontine white matter, a finding consistent with Wallerian degeneration. Immunohistochemical staining for the clone CR3/43 (anti-HLA DP/DQ/DR) shows abundant microglia activation (original magnification in [G]: ×20; in [H]: ×4). CAR = chimeric antigen receptor; H&E = haematoxylin and eosin; HLA = human leukocyte antigen; LFB-PAS = Luxol fast blue with periodic acid-Schiff; neg. DNA = negative control DNA; NTC = no template control; pos. DNA = positive control DNA.

Despite neurologic symptoms being frequently encountered following CAR T-cell infusion, pathogenesis remains not fully understood.^6^ In our patient, we found evidence for blood-brain-barrier dysfunction (as displayed by diffusion restriction on imaging, elevated CSF protein, and autopsy findings) and perivascular infiltration of CD3^+^/CD8^+^-lymphocytes which may represent CAR T-cells as indicated by PCR. Parker et al^7^ recently reported that brain mural cells which are critical for blood-brain-barrier integrity express the CD19-isoform which is recognized by CAR T-cells. Cross-reactivity of CAR T-cells may therefore translate into increased permeability of the brain vasculature; and perivascular lymphoid cells as well as fluid extravasation in patients with fatal neurotoxicity has also been reported by others.^4,8,9^ Moreover, using a murine model, we have previously shown that systemically applied CD19-directed CAR T-cells are able to migrate into the CNS.^10^ In the present case, we also encountered perivascular CD3^+^-lymphocytes staining double negative for CD4/CD8, and such cells may promote neuroinflammation.^11^ These results suggest that on-target-off-tumor activity of CD19-directed CAR T-cells contributes to neurotoxicity, and our case seems to support such a hypothesis. It remains puzzling why the infratentorial brain region was particularly affected in our case.

Following blood-brain-barrier disruption after CAR T-cell infusion, enrichment of proinflammatory cytokines within the CNS has previously been described. Such a mechanism may eventually give rise to glial injury, and finally to clinical symptoms.^12,13^ Accordingly, we found morphological correlates of microglia injury and activation particularly in pontine tissue areas which were predominantly affected from blood-brain-barrier disruptions.^8^ Interleukin-1 was found to be a crucial mediator of neurotoxicity after CAR T-cell therapy, and interleukin-1 blockade may therefore represent a therapeutic avenue.^14^ However, such an approach was not successful in our case. On a cautionary note, no definitive conclusions can be derived from our single case.

Collectively, the presented case highlights the role of blood-brain-barrier dysfunction in neurotoxicity following CAR T-cell therapy. Accumulation of CAR T-cells within the perivascular space and potentially on-target-off-tumor effects may contribute to such a toxicity. Given the risk of fatalities in the context of neurotoxicity, studies on the pathogenesis and treatment algorithms are needed to improve patient care.

## Acknowledgments

All authors thank the patient and her family.

## Sources of funding

PK acknowledges research grants from the Friedrich-Baur-Foundation and from the “Support Program for Research and Teaching” at the Ludwig-Maximilians-University Munich. FS received support from the European Union’s Horizon 2020 Research and Innovation Program (grant agreement MSCA-IF-EF-RI 792832). MS acknowledges support by the Deutsche Forschungsgemeinschaft (SFB 1243 project A10), the Bavarian Elite Graduate School “i-target,” the Else Kröner Fresenius Kolleg “Cancer Immunotherapy,” and the Wilhelm-Sander Stiftung. LvB acknowledges support by the Else Kröner Fresenius Kolleg “Cancer Immunotherapy.”

## Disclosures

JC-T is a Consultant/speaker honoraria from BrainLab and Carthera, and received Royalties from Springer Publisher Intl. MvB-B received research support from Amgen, Astellas, Mologen, Miltenyi, Novartis, MSD, and KITE/Gilead; received honoraria from Astellas, Novartis, MSD, KITE/Gilead. MS received industry research support from Amgen, Gilead, Milteny, Morphosys, Roche, and Seattle Genetics; and has served as a consultant/advisor to Amgen, BMS, Celgene, Gilead, Pfizer, Novartis, and Roche. MS sits on the advisory boards of Amgen, Celgene, Gilead, Janssen, Novartis, Pfizer, and Seattle Genetics, and serves on the speakers’ bureau at Amgen, Celgene, Gilead, Janssen, and Pfizer. The remaining authors have no conflicts of interest to disclose.
